# Candida and Candidiasis

**DOI:** 10.3201/eid0808.020059

**Published:** 2002-08

**Authors:** Mary E. Brandt

**Affiliations:** Centers for Disease Control and Prevention, Atlanta, Georgia, USA

Yeast of the genus *Candida* have exploded into prominence in recent years as opportunistic and nosocomial fungal pathogens. However, the most recent textbook on these organisms was written in 1988. Candida and Candidiasis is a worthy successor in providing comprehensive information on the biology of these organisms.

A total of 28 chapters cover the general properties, virulence factors, cell biology, immunity, genomics, diseases, and laboratory aspects of *Candida* species, with particular emphasis on its most prominent member, *Candida albicans*. The strongest chapters are those covering research aspects of these organisms. Complex subjects like the chemistry of the cell wall, host recognition and adherence, the cell biology of the yeast-hyphal transformation, and extracellular hydrolases as virulence factors in *C. albicans* are well summarized with clear, useful graphics and current references. The book is beautifully laid out, with a series of color plates that help describe phenotype switch variants and chromosome maps.

The clinical chapters appear rather superficial for an infectious diseases clinician but may be useful to a student seeking basic material. The chapter on identification and subtyping contains information available in other sources for less than the cost of this book. A discussion of current practices in antifungal susceptibility testing of *Candida *species would have been helpful. Chapters 2 and 4 contain repetitious material, including photographs of *C. dubliniensis*. A consolidated chapter on the epidemiology of *Candida* infections should be considered for the next edition. The chapters covering the cell biology are most useful, either as a comprehensive overview or as a reference text for researchers and students interested in the biology of these organisms.

